# Immunotherapies in rare cancers

**DOI:** 10.1186/s12943-023-01720-2

**Published:** 2023-02-01

**Authors:** Sneha Vivekanandhan, Deborah Bahr, Ashish Kothari, Mohammed Ali Ashary, Mizba Baksh, Emmanuel Gabriel

**Affiliations:** 1 Department of Immunology, Mayo Clinic, Jacksonville, FL 32224 USA; 2 Department of Microbiology, All India Institute of Medical Sciences, Rishikesh, 249203 India; 3 Department of Surgery, Division of Surgical Oncology, Mayo Clinic, 4500 San Pablo Rd, Jacksonville, FL 32224 USA

**Keywords:** Rare, Cancers, Immune checkpoint inhibitors, PD1, CTLA-4, CAR T cells, Macrophages, Neoantigens, Vaccines, PDL1, Tumor associated macrophages, Dendritic cells

## Abstract

Cancer remains a leading cause of death worldwide, placing a significant burden on healthcare systems as well as the global economy. Rare cancers comprise a group of about 200 cancers that individually occur at extremely low frequencies. In the United States (US), their frequency is approximately 15 cases per 100,000 people, and it is even lower in Europe with approximately 6 cases per 100,000 people. However, combined their frequency of occurrence is much higher than any singular cancer. Cancer treatment and management has tremendously improved in the last decade, particularly with the administration of immune-based therapies. The four most prevalent immune-based therapies are (1) the use of immune-checkpoint inhibitors, (2) macrophage therapy, (3) Chimeric Antigen Receptor (CAR) T cell therapy, and (4) neoantigen-based therapies. In our review, we discuss these various aproaches and their implementation in the treatment of a variety of rare cancers. Furthermore, we discuss their limitations and potential strategies to overcome them to enhance the therapeutic efficacy of these approaches. Finally, our article presents the future directions and other additional immune therapies that may be incorporated into the treatment of rare cancers.

## Introduction

Cancer is the second leading cause of death globally, including in the US. The American Cancer Society has estimated that about 1.9 million new cases were diagnosed in the U.S. in 2022. The most prevalent cancers amongst men are lung, prostate, and colorectal cancers, while women are commonly afflicted with breast, lung, and colorectal cancer (https://www.cancer.org/content/dam/cancer-org/research/cancer-facts-and-statistics/annual-cancer-facts-and-figures/2022/2022-cancer-facts-and-figures.pdf). Conversely, certain cancers such as Merkel cell cancer, types of hepatobiliary cancers, mesothelioma, and adrenal cancers (amongst others) have low incidences and are categorized as rare cancers. The US National Cancer Institute defines rare cancers as those that have less than 15 cases per 100,000 people each year, while the European Union includes cancers that have less than 6 cases per 100,000 people per year (https://www.cancer.gov/). About 200 different cancers are grouped in this category, and together their incidence is higher than any particular cancer [1]. However, the clinical outcome of these cancers is usually grim. These cancers are difficult to diagnose, and often the cancers are identified only after they have progressed into advanced stages. The lower incidences translate into limited samples (biopsy or surgical tissues, patient-derived cell lines) for preclinical studies and few clinical trials to evaluate new interventions or therapeutic strategies. These facts highlight the importance of studying these cancers and identifying the most effective therapeutic strategies to improve patient outcomes.

The conventional treatments administered for both common [2] and rare cancers (https://www.cancer.gov/) include surgery, chemotherapy, radiotherapy and often a multidisciplinary combination of these treatments. The traditional paradigm of these therapies is to target and eliminate the cancer cells by interfering with tumor cell growth and survival. Unfortunately, this limits the efficacy of these treatments as cancers often escape with the help of acquired mutations and cancer stem cells, leading to relapse. Additionally, these therapies usually generate a multitude of harmful side effects. These issues have brought research and clinical focus to immunotherapies. Similar to other health disorders, the immune system, both innate and adaptive, are activated in response to cancer. The immune system plays key roles in both suppressing and promoting cancers by being involved in all aspects of response to cancer: (a) elimination of cancer cells, (b) maintaining equilibrium between tumor cells and immune cells, and (c) facilitating the growth of tumor cells in an immunocompetent host microenvironment [3]. The increased understanding of our immune system and identification of neoantigens has brought attention to identifying and developing strategies to augment immune responses directed towards elimination of these cancer cells and re-activation of anti-tumor responses with the help of memory cells in the event of cancer relapse. In this review, we discuss the various immune-based therapeutic approaches and their current status in the treatment of rare cancers, their limitations and potential strategies to overcome them, recent advances in the identification of biomarkers, and future directions in immunotherapy for rare cancers.

## Immune-based therapeutic approaches and their current status in the treatment of rare cancers

### Immune-checkpoint inhibitors

Immune cells express receptors known as immune checkpoints that are involved in the regulation of immune homeostasis, specifically activation of T cells, certain myeloid cells, and regulatory cytokines. Cancer patients have deficient regulatory systems, wherein immune-checkpoint pathways promoting immune-suppressive functions are upregulated and immune-activating pathways are downregulated [4]. In the past decade, immune-checkpoint inhibitors, predominantly monoclonal antibodies, have positively impacted cancer management and treatment, thereby gaining prominence. Immune-checkpoint inhibitors have been reported to generate sustainable responses and are administered in metastatic and more recently in neoadjuvant and adjuvant settings [5]. One immune checkpoint (IC) receptor, CTLA-4, has shown extensive promise as therapeutic target. CTLA-4, a structural homologue of CD28 is a membrane receptor on cytotoxic T cells [6]. Activation of T cells occurs in two steps, the first being the recognition of antigens presented by the MHC and the second being costimulation generated upon binding of CD28 to CD80 or CD86 on the antigen presenting cells. CTLA-4 can bind to CD80 and CD86 with higher affinity than CD28, thereby impeding T cell activation [7]. CTLA-4 is expressed constitutively on T regulatory (Treg) cells as well as on activated effector T cells [8, 9]. Ipilimumab, an antibody that targets CTLA-4, was the first immune-checkpoint inhibitor to receive approval from the U.S. food and drug administration (FDA) in 2011 for use in cancer treatment. This was in melanoma based on the results of clinical trial NCT00094653 [10]. While in melanoma patients, Ipilimumab demonstrated significant benefit with combined data analysis from 12 trials indicating improved 10-year survival, [11], it has had limited success in other cancers [12].

Another immune-checkpoint target is Programmed Cell Death protein 1 (PD1). PD1 is a key immune-checkpoint receptor that is expressed by T cells and mediates immunosuppression. Its ligand is programmed cell death ligand 1 (PDL1), which is expressed by T cells, B cells, and some non-hematopoietic cells. In a normal immune system it regulates T cell function; however tumors utilize this pathway to their benefit by upregulating PDL1 on their surface and binding to the PD1 on T cells [13]. This interaction causes apoptosis of T cells. Thus, targeting the PD/PDL1 pathway is helpful in targeting tumors. The inhibition of this interaction facilitates normal T cell surveillance and the endogenous anti-tumor response can be increased [14]. Many PD1 inhibitors including nivolumab, pembrolizumab, and cemiplimab, as well as PDL1 inhibitors such as atezolizumab, avelumab, and durvalumab have been approved in recent years [15]. They have shown to be effective and safe for treating melanoma, renal cell carcinoma, and non-small cell lung cancer (NSLC) [16].

Based on the success of immune-check point inhibitors, there are several completed and ongoing clinical trials demonstrating the efficacy of these inhibitors in treating rare cancers. These are summarized in Table 1. Overall, although the use of checkpoint inhibitors is not yet mainstream for rare cancers, there have been several trials reporting their efficacy. Recently, much attention has been turned to different rare malignancies, including non-melanoma cutaneous cancers (including the much less common Merkel cell cancer), hepatobiliary cancers, endocrine and adrenal cancers, and mesothelioma. Active clinical trials are also included in the next paragraphs of this section. For all rare cancers as defined by the National Cancer Institute, a large phase 2 study intending to enroll over 800 patients, is ongoing (NCT02834013 Nivolumab and Ipilimumab in Treating Patients with Rare Tumors). This study investigates nivolumab and ipilimumab in treating patients with rare tumors. For the purposes of this review, however, we focus on the rare solid cancers that have recent clinical trial data within the last decade.

**Table 1:** Ongoing Clinical Studies in Rare Tumors utilizing Immune-checkpoint inhibitors

Cancer	Immune-checkpoint Inhibitor	Trial/Study Category	No. of patients	Pretreated with standard therapies	Outcome	Ref
Metastatic Merkel cell carcinoma	10 mg/kg avelumab by 1-h intravenous infusion every 2 weeks	NCT02155647	88	Yes	ORR was 31.8% (95% CI, 23.3–43.8%; complete response: 11.4%).	[17]
Advanced Merkel Cell Carcinoma	pembrolizumab (2 mg/kg every 3 weeks) for up to 2 years	NCT02267603	50	No	ORR was 56% (complete response [24%] plus partial response [32%]; 95% CI, 41.3 to 70.0%), with ORRs Grade 3 or greater treatment-related adverse events occurred in 14 (28%) of 50 patients and resulted in treatment in seven (14%) of 50 patients, including one treatment-related death.	[18]
Squamous cell carcinoma	200 mg of pembrolizumab intravenously every 3 weeks until disease progression or intolerance	Open-label, phase II trial NCT02721732	19	Yes	At 27 weeks NPR: 36%, ORR 31%, and CBR 38%	[19]
Unresectable cutaneous squamous cell carcinomas	pembrolizumab (200 mg every 3 weeks)	NCT02883556	39-patient primary cohort 18-patient expansion cohort to assess difference between PDL1+ and PDL1-	First-line pembrolizumab monotherapy	Primary cohort’s ORR_W15_ was 41% (95% CI, 26 to 58%) ORR_W15_ for the entire population was 42%; it was significantly higher for PDL1+ patients (55%) versus PDL1- patients (17%; *P* = .02) Pembrolizumab-related adverse events affected 71% of the patients, and 7% were grade ≥ 3. One death caused by rapid CSCC progression; another from a fatal second aggressive head and neck squamous cell carcinoma diagnosed 15 weeks post inclusion	[20]
Advanced cutaneous squamous-cell carcinoma	cemiplimab (3 mg per kilogram of body weight) every 2 weeks a	NCT02383212 NCT02760498	26 59		In phase I study, 13 of 26 responded (50%; 95% confidence interval [CI], 30 to 70). In the metastatic-disease cohort of the phase II study, 28 of 59 patients (47%; 95% CI, 34 to 61) showed response; responses laste4d 6 months in 57% and in 82% until the data cut-off	[21]
Hepatocellular carcinoma	Tremelimumab (300 mg, one dose) plus durvalumab (1500 mg every 4 weeks; durvalumab (1500 mg every 4 weeks); or sorafenib (400 mg twice daily)	NCT05345678	393 patients: tremelimumab plus durvalumab 389 patients: durvalumab 389 patients: sorafenib	Yes	Median OS was 16.43 months (95% CI, 14.16 to 19.58) with tremelimumab plus durvalumab, 16.56 months (95% CI, 14.06 to 19.12) with durvalumab only, and 13.77 months (95% CI, 12.25 to 16.13) with sorafenib.	[22]
Hepatocellular carcinoma	Dose of 200 mg intravenously every 3 weeks among patients	NCT02658019	28	Some patients received sorafenib	One patient had a complete response; 8 patients achieved partial responses for an overall response rate of 32%. Four other patients had stable disease	[23]
Hepatocellular carcinoma (HCC)	15 mg/kg IV of Tremelimumab every 90 days until tumor progression or severe toxicity	NCT01008358	17		no CRs were observed and 3 patients (17.6%) had a confirmed PR that lasted for 3.6, 9.2, and 15.8 months. Objective responses were detected after the first cycle in 1 patient and after the second cycle in the other 2 patients. Ten patients (58.8%) had an SD as the best response to treatment, accounting for a disease control rate of 76.4%	[24]
Hepatocellular carcinoma	200 mg pembrolizumab intravenously every 3 weeks for about 2 years or until disease progression, unacceptable toxicity, patient withdrawal	NCT02702414	104	Yes sorafenib	1% complete and 17 (16%) partial responses; 46 (44%) patients had stable disease, 34 (33%) had progressive disease, and six (6%) patients were not assessable. 76 (73%) of 104 patients, which were serious in 16 (15%) patients	[25]
Hepatocellular carcinoma	Patients were randomly divided in 2:1 ratio to receive either atezolizumab plus bevacizumab or sorafenib	NCT03434379	336 + 135	No prior systemic therapy	Overall survival at 12 months was 67.2% (95% CI, 61.3 to 73.1) with atezolizumaband bevacizumab and 54.6% (95% CI, 45.2 to 64.0) with sorafenib Grade 3 or 4 adverse events occurred in 56.5% of 329 patients who received at least one dose of atezolizumaband bevacizumab and in 55.1% of 156 patients who received at least one dose of sorafenib	[26]
Biliary tract cancers	A combination of gemcitabine 1000 mg/m^2^, cisplatin 75 mg/m^2^ and nivolumab 3 mg/kg was administered every 3 weeks for up to six cycles. Maintenance treatment of gemcitabine and nivolumab was administered to patients achieving disease control following the combination therapy.	NCT03311789	27		15 (55.6%) patients achieved an objective response, including 5 (18.6%) with a complete response (CR), and the DCR was 92.6%.	[27]
Biliary tract carcinomas	Patients were administered durvalumab (1500 mg at day 1 [D1] of each cycle)/tremelimumab (75 mg at D1 for 4 cycles; Arm A) or durvalumab/tremelimumab with paclitaxel (80 mg/m^2^ at D1, D8, D15; Arm B) every 28 day	NCT03704480	20		No dose-limiting toxicities (DLT) were observed in Arm A; 50% of patients in Arm B had DLT	[28]
Biliary tract carcinomas	240 mg of Nivolumab administered intravenously every 2 weeks for 16 weeks, and then 480 mg was administered intravenously every 4 weeks until disease progression or unacceptable toxic effects occurred	NCT02829918	54	Yes	Objective response determined was 22% (10 of 46), including 1 unconfirmed partial response, with a disease control rate of 59% (27 of 46). Central independent review determined objective response rate to be 11% (5 of 46), including 1 unconfirmed partial response, with a disease control rate of 50% (23 of 46).	[29]
Biliary tract carcinomas	3 mg/kg of nivolumab and 1 mg/kg of ipilimumab every 3 weeks for 4 doses, followed by nivolumab 3 mg/kg every 2 weeks and continued for up to 96 weeks until disease progression	NCT02923934	39	Some had chemotherapy	The ORR was 23% (*n* = 9) with a disease control rate of 44% (*n* = 17) Responders were patients with intrahepatic cholangiocarcinoma and gallbladder carcinoma. 49% of patients (*n* = 19) had immune related toxic effects of which 15% (*n* = 6) were grade 3 or 4	[30]
Adrenocortical carcinoma	200 mg of pembrolizumab intravenously every 3 weeks until disease progression or intolerance		15	Yes	NPR at 27 weeks was 31%, ORR 15%, and CBR 54%	[19]
Adrenocortical carcinoma	nivolumab (3 mg/kg IV) and the anti-CTLA-4 antibody ipilimumab (1 mg/kg IV) every three weeks for four doses, followed by nivolumab (3 mg/kg IV) every two weeks and continued for up to 96 weeks		6	Yes	PR 33%, SD 33%	[31]
Paraganglioma–pheochromocytoma	200 mg of pembrolizumab intravenously every 3 weeks until disease progression or intolerance		9	Yes	NPR at 27 weeks was 43%, ORR 0%, and CBR 75%.	[19]
Pleural mesothelioma	Patients were administered nivolumab (240 mg every 2 weeks) and ipilimumab (1 mg/kg every 6 weeks up to four times) intravenously. Treatment was given up to 2 years or until disease progression or unacceptable toxicity. The primary endpoint was disease control at 12 weeks	NCT03048474	34	Yes	10 (29%) patients had a partial response and 13 (38%) patients had stable disease; thus, disease control was achieved in 23 (68, 95% CI 50–83) of 34 patients.	[32]
Malignant mesothelioma	Patients were randomly assigned into tremelimumab (*n* = 382–2) or placebo (*n* = 189) groups and administered intravenous tremelimumab (10 mg/kg) or placebo every 4 weeks for 7 doses and every 12 weeks thereafter until a treatment discontinuation criterion was reached. The primary endpoint was overall survival in the intention-to-treat population.	NCT01843374	569	Yes	307 (80%) of 382 patients the tremelimumab group and 154 (81%) of 189 patients in the placebo group died. Median overall survival was not different between groups. Treatment-related adverse events of grade 3 or worse occurred in 246 (65%) of 380 and and 91 (48%) of 189 patients in the tremelimumab and placebo group respectively. Treatment-caused death occurred in 36 (9%) of 380 patients in the tremelimumab group and 12 (6%) of 189 in the placebo group.	[33]
Malignant mesothelioma	10 mg/kg tremelimumab was administered once every 4 weeks for six doses, then every 12 weeks until disease progression, unacceptable toxic effects, or treatment discontinuation	NCT01655888	29	Yes, platinum-based regimen	After a median follow-up of 21·3 months four immune-based-partial responses were observed, one at the first tumor assessment (after about 12 weeks) and three at the second tumor assessment (about 24 weeks), with two responses occurring after initial progressive disease and one response after initial stable disease. Grade 3–4 adverse events occurred in two (7%) patients	[34]
Mesothelioma	cisplatin 75 mg/m^2^, pemetrexed 500 mg/m^2^, and durvalumab 1125 mg intravenously on day 1 of a 3-weekly schedule for a maximum of six cycles. Change from cisplatin to carboplatin with an area under the curve of 5 was permitted. Durvalumab was continued for a maximum of 12 months. The primary endpoint was progression-free survival at 6 months.	Australia New Zealand Clinical Trials Registry, ACTRN12616001170415	54	Concurrent chemotherapy	31 (57%) of 54 patients were alive and progression-free at 6 months.	[35]
Malignant mesothelioma	Single agent pembrolizumab 200 mg every 3 weeks until completion of 35 cycles		118	No	OR of 8%	[36]
Malignant mesothelioma	durvalumab 1120 mg once every 3 weeks in combination with pemetrexed and cisplatin (standard doses) for up to six cycles	NCT02899195	55	Yes	durvalumab plus platinum-pemetrexed chemotherapy had a median OS of 20.4 months versus 12.1 months (historical control)	[37]
Malignant mesothelioma	Nivolumab (3 mg/kg every 2 weeks) plus ipilimumab (1 mg/kg every 6 weeks) for up to 2 years compared to 6 cycles of platinum plus pemetrexed chemotherapy.		605 303 Nivolumab plus ipilimumab 302 chemotherapy (platinum plus pemetrexed)	No	For the nivolumab plus ipilimumab group compared to chemotherapy (platinum plus pemetrexed):Median OS was 18.1 versus 14.1 months [HR 0.73 (95% CI 0.61–0.87)] 3-year OS rates were 23% versus 15%. 3-year PFS rates were 14% versus 1%. ORR were 40% versus 44%.	[38]
Malignant mesothelioma (Peritoneum)	Atezolizumab 1200 mg in combination with bevacizumab 15 mg/kg intravenously every 21 days		20	Yes – bevacizumab	OR of 40%. 1-year PFS was 61%, and 1-year OS was 85%	[39]
Small cell malignancies of non-pulmonary origin	200 mg of pembrolizumab intravenously every 3 weeks until disease progression or intolerance		11	Yes	Ongoing	[19]
Metastatic sarcoma	Intravenous nivolumab 3 mg/kg every 2 weeks, or nivolumab 3 mg/kg + ipilimumab 1 mg/kg every 3 weeks for four doses. All patients received nivolumab monotherapy (3 mg/kg) every 2 weeks for up to 2 years thereafter	NCT02500797	43 patients: nivolumab monotherapy 42 patients: nivolumab plus ipilimumab		Endpoint responses were observed two (5% [92% CI 1–16] of 38 patients) in the nivolumab group and six (16% [7–30] of 38 patients) in the combination group. Eight (19%) of 42 patients receiving monotherapy and 11 (26%) of 42 patients had Serious treatment-related adverse events.	[40]
Medullary renal cell carcinoma	200 mg of pembrolizumab intravenously every 3 weeks until disease progression or intolerance		4	Yes	Ongoing	[19]
Vascular sarcoma	200 mg of pembrolizumab intravenously every 3 weeks until disease progression or intolerance		7	Yes	Ongoing	[19]
Penile carcinoma	200 mg of pembrolizumab intravenously every 3 weeks until disease progression or intolerance		3	Yes	Ongoing	[19]
Germ cell tumor	200 mg of pembrolizumab intravenously every 3 weeks until disease progression or intolerance		12	Yes	Ongoing	[19]
Carcinoma of unknown primary	200 mg of pembrolizumab intravenously every 3 weeks until disease progression or intolerance		22	Yes	NPR at 27 weeks was 33%, ORR 23%, and CBR 54%	[19]

Kaufman et al [17] performed a phase 2 trial of avelumab (anti-PD1 antibody) in patients with metastatic Merkel cell cancer who had failed chemotherapy. An overall response rate of 31.8% was reported, with minimal risk of adverse events (6%). Nghiem et al [41] reported an even higher response rate of 56% is a similar population of advanced Merkel cell cancer patients. Interestingly, responses were observed in both patients with Merkel-cell polyomavirus (MCPyV) positive tumors and negative tumors, suggesting a common immune pathway between both subtypes of Merkel cell cancer. The potential role of checkpoint blockade in the adjuvant setting is actively being investigated in several clinical trials as well, including NCT04291885 Immunotherapy Adjuvant Trial in Patients with Stage I-III Merkel Cell Carcinoma (I-MAT), NCT03271372 Adjuvant Avelumab in Merkel Cell Cancer (ADAM), NCT03798639 Nivolumab and Radiation Therapy or Ipilimumab as Adjuvant Therapy in Treating Patients with Merkel Cell Cancer, and NCT03712605 Testing Pembrolizumab versus Observation in Patients with Merkel Cell Carcinoma After Surgery, STAMP Study. Due to the aggressive nature of Merkel cancer and the failure of distant disease control often leading to patient mortality, these new trials offer new adjuvant treatment for this rare but highly morbid cancer. Other studies for non-melanoma skin cancers are also listed in Table 1, including the more common squamous cell cancer.

Hepatobiliary cancers are often very morbid cancers with limited treatment options, and as such, many investigators have tested checkpoint blockade in these patients in the hopes that durable responses can be achieved. A recent meta-analysis analyzing this pathway for hepatocellular carcinoma showed that a high expression of PDL1 was significantly associated with a poor overall survival rate, which demonstrates the pathway’s prominent role in tumor progression [42]. However, current therapeutic studies are limited, and the results of these studies have not been very promising, as shown in Table 1. Overall, the numbers of patients in these studies have been relatively low with limited follow-up, low complete response rates, and modest benefits over standard over care (e.g., chemotherapy or targeted therapy). One of more recent studies by Kelley et al [22] tested the combination of tremelimumab (anti-CTLA4) with durvalumab (anti-PD1) compared to durvalumab alone or sorafenib (as standard of care). Compared to sorafenib, durvalumab alone or in combination with tremelimumab resulted in an increase in median OS of about 2–3 months (median OS 16.43 months, 95% CI 14.16 to 19.58) with tremelimumab and durvalumab and 16.56 months (95% CI, 14.06 to 19.12) with durvalumab alone, compared to 13.77 months (95% CI, 12.25 to 16.13) with sorafenib. It is thought that the immunologically “cold” microenvironment of hepatobiliary tumors contributes to relatively poor responses to immunotherapy [43, 44]. Active clinical trials seek to combine immunotherapy with ablative techniques in order to produce antigen targets for immunotherapy and may hold promise for future discovery. These include NCT03101475 Synergism of Immunomodulation and Tumor Ablation (ILOC), NCT03937830 Combined Treatment of Durvalumab, Bevacizumab, Tremelimumab and Transarterial Chemoembolization (TACE) in Subjects with Hepatocellular Carcinoma or Biliary Tract Carcinoma, NCT04605731 Durvalumab and Tremelimumab after Radioembolization for the Treatment of Unresectable, Locally Advanced Liver Cancer, NCT05301842 Evaluate Durvalumab and Tremelimumab +/− Lenvatinib in Combination with TACE in Patients with Locoregional HCC (EMERALD-3), and NCT04522544 Durvalumab (MEDI4736) and Tremelimumab in Combination With Either Y-90 SIRT or TACE for Intermediate Stage HCC With Pick-the-winner Design.

Another rare solid cancer for which there has been recent progress with immunotherapy is adrenal tumors. Like hepatobiliary cancers, adrenal cortical carcinoma (ACC) is associated with a dismal prognosis, and there are limited viable treatment options. Naing et al [19] and Klein et al [31] reported modest efficacy of single agent (pembrolizumab) or dual agent (nivolumab and ipilimumab), respectively, for patients with advanced ACC. These trials had small cohorts of 15 or less patients. Within the study by Naing et al, the investigators reported similarly modest results for adrenal pheochromocytomas or paragangliomas (as shown in Table 1). As one of the rarest cancers, few active clinical trials are investigating immunotherapy for ACC, and these include NCT00457587 Preclinical Study Towards an Immunotherapy in Adrenocortical Carcinoma and NCT02673333 Single Agent Pembrolizumab in Subjects with Advanced Adrenocortical Carcinoma.

Lastly, immunotherapy has recently been approved as first-line therapy for pleural malignant mesothelioma. Early trials in the mid to late 2010s showed modest response rates to tremelimumab [33, 34]. However, larger randomized clinical trials in published in the 2020s showed superior benefit with other checkpoint inhibitors. CheckMate 743 randomized patients to nivolumab plus ipilimumab versus traditional chemotherapy with cisplatin and pemetrexed. All outcomes were improved with immunotherapy compared to chemotherapy, with an increase in 3-year OS rates (23% versus 15%) and 3-year PFS (14% versus 1%) [38]. Similarly, PrE0505, which was a phase 2, single-arm, multicenter study, enrolled patients with previously untreated pleural mesothelioma reported an 8-month OS benefit with durvalumab plus cisplatin and pemetrexed compared to historical chemotherapy only controls [37]. While the majority of studies with mesothelioma have been performed for pleural-based disease, its use is being investigated in the less common peritoneal mesothelioma. Raghav et al [39] recently published their small cohort of 20 patients with peritoneal mesothelioma treated with atezolizumab in combination with bevacizumab, achieving an OR of 40%, 1-year PFS of 61%, and 1-year OS of 85%. Intuitively, the biology of peritoneal mesothelioma may behave similarly to pleural mesothelioma, so these latest results are encouraging for the rarer peritoneal-based disease. Ongoing clinical trials in peritoneal mesothelioma may contribute to the growing body of evidence that immunotherapy may be effective for this site of disease as it is for pleural-based disease. These trials include NCT05001880 Chemotherapy with or without Immunotherapy for Peritoneal Mesothelioma and NCT05041062 A Study of Immunotherapy Drugs Nivolumab and Ipilimumab in Patients with Resectable Malignant Peritoneal Mesothelioma.

In summary, the clinical application and utility of immunotherapy for rare cancers has been mixed in recent years, with significant improvement in outcomes for certain cancers (Merkel cell carcinoma and pleural/peritoneal mesothelioma), but less encouraging for others (hepatobiliary cancers and endocrine/adrenal malignancies). The biology and microenvironment as well as tumor vessel heterogeneity among the distinct types of tumors may account for differences in response among solid tumors [45]. Ongoing and future studies that combine immunotherapy with other treatment modalities (including the clinical trials listed throughout this section) may become a valid option for treatment-refractory patients with rare cancers. The CRAFT trial is one example of how individualized targeted therapy combined with immunotherapy (anti-PDL1) may augment responses by addressing both actionable genetic targets and the tumor immune microenvironment [46]. Innovative clinical trials such as these may achieve higher and more durable responses for patients with rare cancers, and the results of these trials are eagerly awaited.

### Macrophage therapy

**Fig. 1:**
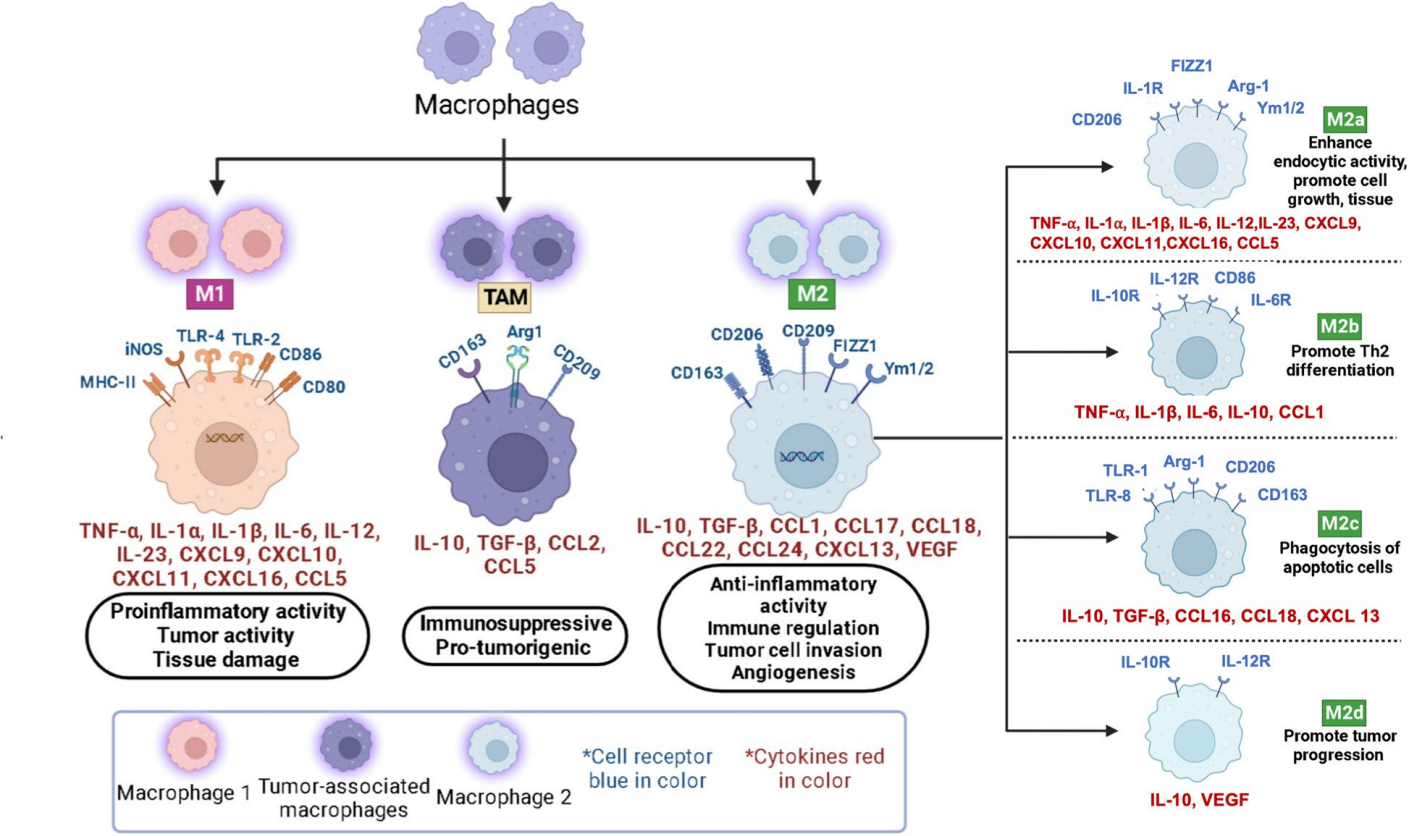
The role of macrophages in tumor growth and progression. Macrophages are involved in several processes associated with tumor growth and progression, including inflammation, immune regulation, angiogenesis, invasion, and metastasis in the solid tumor microenvironment. Each subtype of macrophage is characterized by the expression of specific cytokines, chemokines, and toll-like receptors

In recent years, a new form of immunotherapy targeting and modulating macrophages has been investigated. Macrophages (Fig. 1) are specialized to their host tissues and perform a variety of functions, including ingesting and degrading dead cells and debris, eliminating pathogens, and regulating inflammatory responses [47]. Traditionally, there are two categories of macrophages, the classically activated macrophages (M1) and the alternatively activated macrophages (M2). M1 macrophages promote inflammatory responses by secreting cytokines such as TNFα, IL1-B, and IL12 to enhance the recruitment of Th1 T cells to the site of inflammation [48]. Additionally, they upregulate genes and other co-stimulatory molecules that enhance T cell response(s), which serve a critical anti-tumor role [49]. On the other hand, M2 macrophages have a role in normal immune function and homeostasis, such as stimulating Th2 cells, eliminating parasites, wound healing, immune regulation, and tissue regeneration as well as the maintenance of the tumor microenvironment (TME) [50].

Like other immune cells, macrophages are found in cancer tissues as well. These are known as tumor-associated macrophages (TAMs). The TAM pool is generated through both monocyte recruitment and through the local/tissue resident macrophage proliferation [51]. The phenotype of TAMs is similar to that of M2 macrophages. They promote tumor progression through increased gene instability, angiogenesis, fibrosis, immunosuppression, lymphocyte exclusion, invasion, and metastasis. Furthermore, they suppress the anti-tumor immunity by inhibiting normal T cell function, including both cytotoxic T cells and Treg cells [52]. TAMs are known to support angiogenesis in two ways – first by promoting angiogenesis initiation in avascular areas and second by helping in the vascular flow through the remodeling of the vasculature [53]. TAMs have been reported to support metastasis by facilitating tumor cell invasion and migration [54]. Thus, macrophages-based immunotherapy has become an increasingly viable option.

Genetically engineered macrophages (GEM) are one platform of macrophages-based immunotherapy. Macrophages can be engineered to modulate the tumor microenvironment to a more anti-tumor one. They can be engineered to secrete proteins like soluble TGF beta-receptor II or interleukin 21 to decrease immune suppression or activate immune cells, respectively. Conversely, they can be engineered to prevent macrophage mediated immune suppression by knocking out the genes involved in the deregulation of cytotoxic cells like PDL1 and interleukin 10 with the help of CRISPR technology [55]. Preclinical data in glioblastoma (GBM) showed promising results with no increased risk to morbidity in animals or increased tumor growth [55]. The encouraging data from these studies warrant further investigation to extend this approach into clinical settings.

Another approach to modulate TAMs is to impair their functioning through antibodies that target proteins expressed on TAMs. Data from a phase I clinical study in 63 diffuse type tenosynovial giant cell tumor patients treated with emactuzumab showed favorable responses. Biopsy tissues were available for 36 patients, and these demonstrated a significant decrease in CSF1R+ and CD68/CD163+ macrophages. Independently the overall objective response rate (ORR) was high at 71%. Additionally, the responses were durable with an ORR of 70 and 64% after one and 2 years post enrollment into the study, respectively [56].

A third approach to macrophage modulation, chimeric antigen receptor (CAR)-T cell-mediated TAM modulation was recently described in an ovarian cancer pre-clinical model. The authors used CAR-T cells to selectively delete folate receptor β expressing (FRβ^+^) TAMs in syngeneic tumor mouse model. This resulted in the enrichment of pro-inflammatory monocytes, increase in tumor-specific CD8^+^ T cells, slowed tumor progression and increased survival [57]. This is an exciting, innovative approach to modulate macrophages using CAR-T cells.

Macrophages have the ability to penetrate and survive within the tumor tissues. Based on this, University of Pennsylvania researchers recently described a new macrophage-based therapy. This is an individualized approach where monocytes are isolated from the patient’s blood, modified with the desired antigen-specific chimeric receptor, and then given back to patients [58]. The FDA recently granted Fast Track designation to a CAR-M, CT-0508, a human epidermal growth factor receptor 2 (HER2) targeted chimeric antigen receptor macrophage for the treatment of patients with solid tumors (https://carismatx.com/carisma-therapeutics-announces-u-s-food-and-drug-administration-grants-fast-track-designation-to-ct-0508-for-the-treatment-of-patients-with-solid-tumors/). This approach, if successful, would be extremely beneficial to other cancers, particularly those in which tumor microenvironment limits the efficacy.

### Chimeric antigen receptor (CAR) T cell therapy

CAR-T cell therapy is a more contemporary form of immunotherapy. T cells are genetically modified to express chimeric receptors encoding an antigen-specific single-chain variable fragment and various stimulatory molecules. Upon administration, these modified T cells traffic to and recognize cancer cells in an HLA-independent manner. T cells expressing CARs have been propelled to the forefront of experimental cell therapies due to their clinical success for hematological malignancies targeting CD22, CD30, and CD-19–expressing B-cell acute lymphocytic leukemia [59–63]. Earlier this year (2022), the US FDA approved CAR-T cell therapies for certain rare cancers, including follicular lymphoma, B-cell non-Hodgkin lymphoma, B-cell acute lymphoblastic leukemia, and Mantle Cell lymphoma [64]. While these developments show tremendous promise in this form of therapy, it is important to note that CAR-T cell therapies for solid tumors have shown limited anti-tumor activity in early phase clinical testing despite targeting a variety of target antigens and tumor types [65–70]. A study in ovarian cancer model demonstrated an RNA vaccine-based approach of increasing the efficiency of CAR-T cells in solid tumors and utilized protein claudin 6 (CLDN6) as the CAR target. CLDN6 is a tight junction that is regulated developmentally. This study showed that delivery of CAR antigens using nanoparticulate RNA vaccine into the lymphoid compartments stimulated the adoptively transferred CAR-T cells. This system promoted the selective expansion of CAR-T cells and tumor regression was achieved at subtherapeutic doses of the CAR-T cell [71]. A phase I/IIa, FIH, open-label, multicenter, clinical trial (NCT04503278) is evaluating the safety and efficacy of CLDN6 CAR-T with or without CLDN6 RNA-LPX in patients with CLDN6-positive relapsed or refractory advanced solid tumor. In Table 2, we have summarized on-going clinical trials evaluating the efficacy of CAR-T cell therapy in rare cancers.

**Table 2:** Ongoing Clinical Studies in Solid Tumors utilizing CAR T-cell therapy

Antigen	Cancer/participants	Interventions	Clinical Stage	Brief Description of Study	CT ID
B7-H3	Recurrent solid tumors including brain tumors, Ewing’s sarcoma (PNET) (100 participants)	Biological: 4SCAR-276	1/2	T cells genetically modified with a 4th generation lentiviral chimeric antigen receptor targeting CD276 (B7-H3).	NCT04432649
EGFR806	Germ Cell Tumor, Retinoblastoma, Hepatoblastoma, Wilms Tumor. (36 participants)	Biological: second-generation 4-1BBζ EGFR806-EGFRt	1	Genetically modified chimeric antigen receptor or CAR) that will target and kill solid tumors.	NCT03618381
GPC3	Hepatocellular carcinoma (10 participants)	Genetic: GAP T cells, Drug: Cytoxan	1	T cells genetically engineered with a GPC3-CAR (GAP T cells) in patients with GPC3-positive solid tumors	NCT02932956
Mesothelin	Mesothelin Positive Multiple Solid Tumors including PC, cholangiocarcinoma and ovarian cancer (10 participants)	Biological: Mesothelin-directed CAR-T cells	1	CRISPR-Cas9 to knocked out the PD1 of the chimeric antigen receptor (CAR) T cells with the combination of Pretreatment by Paclitaxel.	NCT03747965
LMP1	Nasopharyngeal (20 participants)	Other: CAR-T cells	1	Determine the efficacy of LMP1-CAR -T cells in the treatment of EBV associated malignant tumors.	NCT02980315
FR-α	Ovarian (50 participants)	Biological: MOv-gamma chimeric receptor gene	1	Interleukin-2 plus gene-modified white blood cells in treating patients who have advanced ovarian epithelial cancer	NCT00019136
DR5	Hepatoma (73 participants)	Biological: CAR-T cell immunotherapy	1/2	CAR-T/TCR-T cell immunotherapy in treating with different malignancies patients.	NCT03638206
c-MET	Breast, hepatocellular (77 participants)	Biological: T cells modified with RNA anti -cMET CAR	1	Intravenously administered, RNA electroporated autologous T cells expressing MET chimeric antigen receptors	NCT03060356
AFP	Hepatocellular carcinoma, liver (3 participants)	Biological: autologous ET1402L1-CART cells	1	ET1402L1-CART-cells in patients with AFP+ HCC	NCT03349255
Claudin 18.2	Advanced solid tumor including advanced gastric cancer, esophagogastric gastroesophageal junction cancer, and pancreatic cancer (123 participants)	Drug: CAR-CLDN18.2 T-Cells, Drug: PD1 Monoclonal Antibody	1	Safety, efficacy, and pharmacokinetics of autologous humanized anti-claudin18.2 chimeric antigen receptor T cell in advanced solid tumor.	NCT03874897
CD70	Metastatic Tumor including Renal Cell Carcinoma, Ovarian Cancer and Cervix Cancer (36 participants)	Biological: CD70 CAR-T cells	1	CAR-T in patients with CD70-positive advanced/metastatic solid tumors, and to obtain the maximum tolerated dose of CAR-T and phase II Recommended dose.	NCT05420545
LCAR	Claudin18.2-positive advanced solid Tumors including advanced gastric cancers (64 participants)	Biological: LCAR-C18S cells	1	Cell-based LCAR-C18S (hereinafter “LCAR-C18S”) in subjects with Claudin18.2-positive advanced solid Tumors.	NCT04467853
NKG2D	Solid Tumors including ovarian, cholangiocarcinoma, and colorectal cancer. (18 participants)	Biological: KD-025	1	Effectiveness of NKG2D-based CAR-T cells infusion in the treatment of advanced NKG2DL+ solid tumors.	NCT05382377
IM96	Digestive System Neoplasms (19 participants)	Drug: IM96 CAR-T cells	1	Efficacy and safety of IM96 CAR-T Cells in Patients With Advanced Digestive System Neoplasms	NCT05287165

**Fig. 2:**
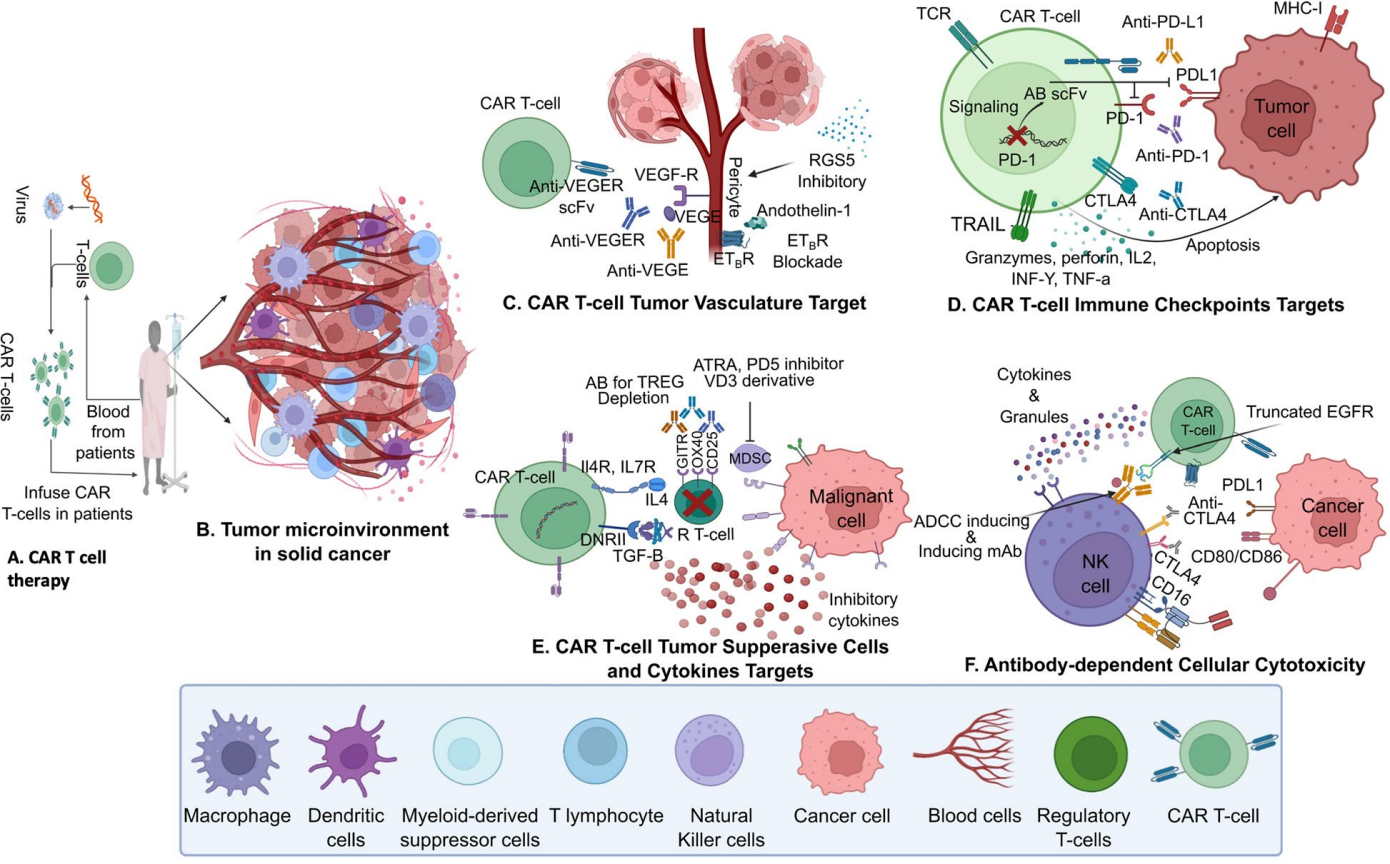
CAR-T cells mediating their anti-tumor effects. CAR-T cells require trafficking to the tumor cell surface so that they may bind to the target molecule to mediate anti-tumor effects. The main targets include immune checkpoints, chemokine-receptor network, tumor vasculature, and immune suppressive cells and cytokines (Fig. 2B-F)

CAR-T cells require trafficking to the tumor cell surface so that they may bind to the target molecule (main targets include immune checkpoints, chemokine-receptor network, tumor vasculature, and immune suppressive cells and cytokines, as shown in Fig. 2B-F). However, the tumor microenvironment impedes this transit. Solid tumors produce chemokines like CXCL1, CXCL12, and CXCL5 within the tumor microenvironment, preventing the T cells from reaching the tumor cells. For example, a study in pancreatic cancer (PC) model, reported that carcinoma-associated fibroblasts (CAF) that expressed fibroblast activation protein (FAP) produced CXCL12. The study showed that the T cell population was less abundant in the regions where FAP+ cells were present, suggesting a link between CXCL 12 expression and T cells [72]. Another study in prostate cancer model showed that CXCL5 secreted by the tumor recruited CXCR2-expressing myeloid-derived suppressor cells (MDSCs) to the tumor microenvironment. This resulted in the secretion of cytokines and enzymes that suppressed the proliferation and the activation of the local T cells [73]. The findings from these relatively common tumor models (PC and prostate) are important as they could be extended to identify potential biomarkers as well further challenges and opportunities in translating CAR-T therapy into the clinic for rare cancers.

Collectively, the data thus far indicate that CAR-T therapy is effective for treating certain rare cancers and its efficacy for some more is currently being evaluated through clinical trials. The identification of biomarkers to predict the sensitivity of the tumor to this therapy, and other molecules whose concurrent therapeutic targeting can enhance the clinical outcome of CAR-T cell therapy would quicken the development of this therapy for a broader range of tumors.

### Neoantigen-based therapies

Neoantigens are tumor-cell specific proteins resulting from mutations in the protein-coding regions of DNA through acquired mutations, alternative splicing and gene rearrangement [74]. Additionally, in most human tumors without a viral aetiology, tumor neoantigens could emanate from an assortment of non-synonymous genetic alterations, including single-nucleotide variants, insertions and deletions, gene fusions, frameshift mutations, and structural variants [75, 76]. Furthermore, cancer-specific mutations often generate neoepitopes which are present on the surface of cancer cells by MHCs. Notably, these may also function as neoantigens [77].Tumor-specific antigen (neoantigen) are usually located on the outer surface of tumor cell and are particularly identified by neoantigen-specific T cell receptors with the help of histocompatibility complexes (MHCs) molecules [75, 76, 78–80]. Some neoantigens enhance therapeutic efficacy and could potentially serve as biomarkers to predict patient response to cancer immunotherapy [78, 81]. Recent literature indicate that neoantigens play a pivotal role in tumor-specific T cell-mediated anti-tumor immunity [79, 80, 82]. Some investigations indicate that neoantigen-targeting approaches can generate strong and durable anti-tumor immune reactions in individual tumor microenvironments. The main neoantigen-based tumor therapies include long synthetic peptide (SLP) vaccines, DNA/mRNA vaccines, dendritic cell-based vaccines, neoantigen-specific T cell receptor-based therapies, and bispecific antibodies associated with public neoantigens. Recent studies have demonstrated the effectiveness and feasibility of neoantigen-targeted cancer vaccines on murine tumor models including oesophagal squamous cell carcinoma [83], glioma [84], and sarcoma [85]. Some of the neoantigen based therapeutic approaches are discussed below.

#### Neoantigen-based adoptive cell therapy (ACT)

This therapy aims at stimulating the patients’ immune response(s) by transferring neoantigen-targeting lymphocytes into the patients. Some studies have reported that the tumor-infiltrating lymphocytes (TILs) in the patients recognize neoepitopes expressed by the patient’s own tumor, underscoring the biological relevance of this therapy. For example, a study reported that adoptive transfer of CD4+ T helper 1 (Th1) cells that recognized a mutation expressed by the tumor cells could mediate the regression of metastatic cholangiocarcinoma [86]. Tran and colleagues [87] reported that in a cohort of 10 patients with metastatic gastrointestinal (GI) cancer, 9 had CD4^+^ and/or CD8^+^ TILs that recognized one to three neoepitopes generated by somatic mutations in the patients’ tumors. Additionally, they reported that these epitopes were unique to the individual patients. However, one of the patients expressed a human leukocyte antigen T cell receptor from CD8^+^ TILs that targeted the KRAS^G12D^ [87]. Similarly, Cafri and colleagues [88] reported the presence of CD4^+^, and CD8^+^ memory T cells that targeted oncogenic KRAS in the peripheral blood of 3/6 metastatic colon cancer patients. Oncogenic KRAS mutations are common in several cancers including GI. Theoretically, expanding or generating these CD8^+^ TILs that target the mutant KRAS and using them for therapy would lead to the development of personalized therapies for a broad spectrum of patients who have tumors with oncogenic KRAS.

Recent studies have identified neoantigen-specific CD8^+^ and CD4^+^ lymphocytes in patients with relatively low tumor mutation burden cancers such as ovarian (naïve; no prior immunotherapy) [89] and gastrointestinal cancers [88, 90]. The identification of neoepitopes directly in the patient samples brings us closer to clinical application by eliminating the developmental processes such as neoantigen prediction and experimental validation; and increases the chances of therapeutic success. Collectively, these findings indicate that neoantigen-specific lymphocytes could be developed as a personalized therapy for cancer.

#### Neoantigen-based vaccines

**Fig. 3:**
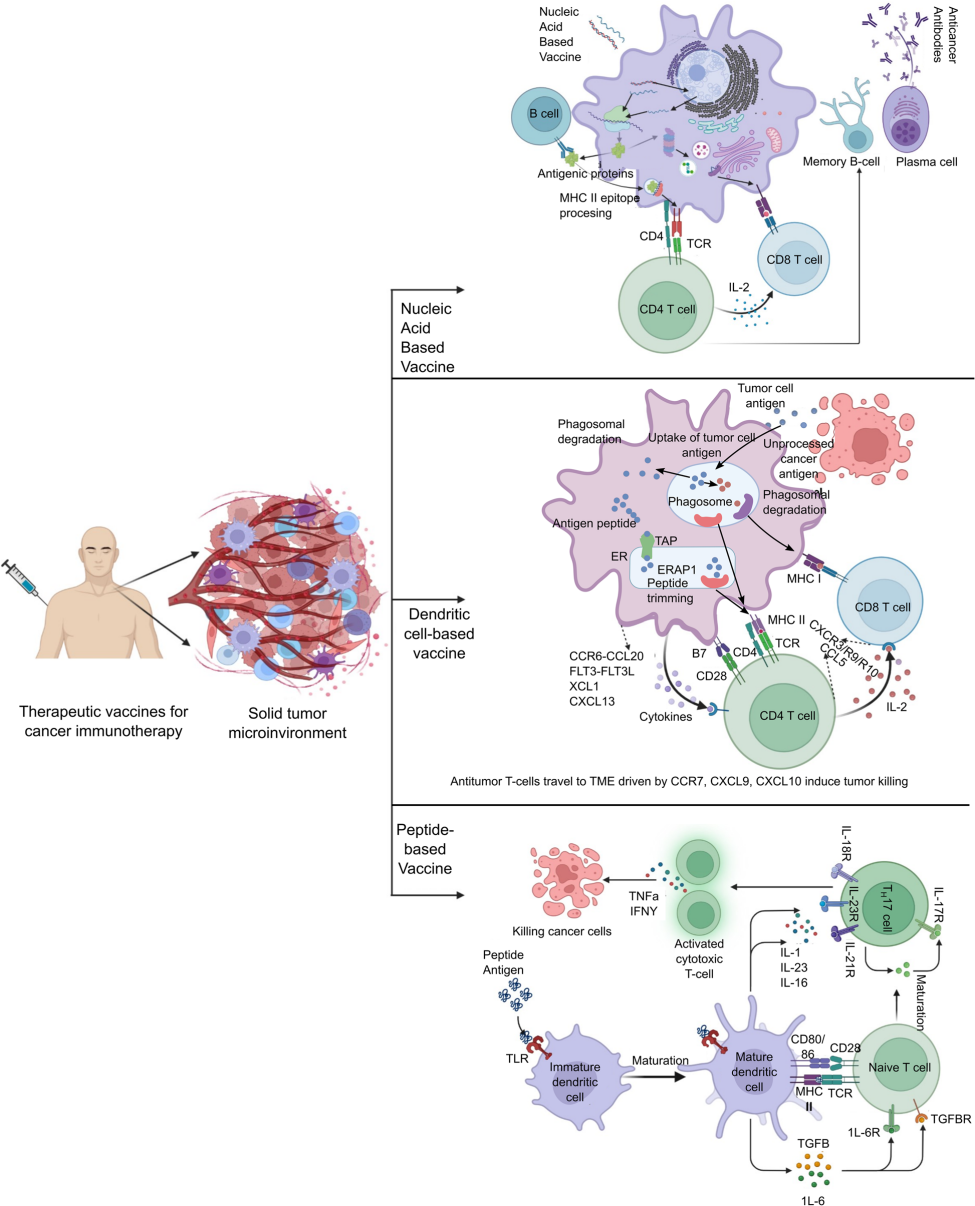
The different type of vaccines being evaluated for cancer immunotherapy. Three major types of vaccines being evaluated for therapeutic targeting are nucleic acid, dendritic cell and peptide based

This is another approach targeting neoantigens in the tumors. Three types of neoantigen-targeting vaccines such as nucleic acid (RNA, DNA) – based, synthetic and dendritic cell (DC) based (Fig. 3) are being evaluted for potential use in clinical settings. A phase 1 clinical study, NCT03313778, examined the neoantigen-based lipid-encapsulated vaccine mRNA-4157 in patient with solid tumors including bladder urothelial carcinoma and human papillomavirus-negative head and neck squamous cell carcinoma along with a few common cancer types. Out of 79 individuals treated with mRNA-4157, 16 were treated as a single treatment and 63 were administered mRNA-4157 with the immune checkpoint inhibitor pembrolizumab. mRNA-4157 was safe and well tolerated. Furthermore, these RNA/DNA based vaccines can be administered through encapsulation by delivery vehicles such as lipid nanoparticles (LNPs) or even orally [91]. These studies suggest that RNA/DNA based vaccines hold tremendous potential for therapeutic use.

Neoantigen based peptide vaccines are yet another form of immune therapy. An effective vaccine would induce a significant T cell response that would effeciently target the tumor cells. Additionally, the CD8+ memory T cells need to be activated for a sustainable respone, in case the cancer relaspses. Emerging data have encouraging data. A a phase 2 study assessed the clinical efficacy of synthetic long-peptide vaccine against the HPV-16 oncoproteins E6 and E7 in women with HPV-16–positive, grade 3 vulvar intraepithelial neoplasia. The patients were vaccinated three to four times. It was reported that the vaccine generated T cell responses in all patients; those who had a complete response in 3 months had a considerably stronger interferon-γ–related CD4+ T cell proliferative and a broader CD8+ interferon-γ T cell response than patients who did not have a complete response. Three months post the last vaccination, 60% patients had a favorable clinical response consisting of complete regression was seen in five women and in four of them HPV was undetectable. Twelve months post the vaccination, clinical response was reported in 79% of the patients with complete responses in 9 out of 19 patients. Additionally, at the 24-month folow up, the complete response rate was still maintained [92].

## Limitations of these immune therapies and potential strategies to overcome them

Immunotherapeutic approaches have undoubtedly significantly improved cancer management and patient outcomes. However, these approaches have some limitations, on pre-clinical, clinical, economic, and social fronts.

A major roadblock in identifying, developing, and evaluating therapies particularly for rare cancers is that only limited samples are available (https://www.cancer.gov/). The incidence of these cancers is low, thereby the tissue samples to assess biomarkers; patient-derived cell lines and xenograft models to research to study underlying mechanisms as well as the ability to conduct clinical trials is very restricted. An article recently proposed building patient-derived rare cancer models by establishing positive collaborations between multiple clinical and research organizations [1]. The success of such a model would theoretically tremendously benefit and accelerate the identification of biomarkers and drugs for the rare cancers. Of note, immunotherapies have heterogeneous therapeutic outcomes with the responses generated being considerably varied between different individuals. Hence, the data generated with the help of such models might be helpful only for a portion of the patients. Identification of reliable biomarkers would likely strengthen these predictions and possibly help identify or streamline the population that would either benefit or be non-responders to these therapies.

In the clinical settings, the challenges are similar to conventional cancer therapies. Several patients acquire resistance to IC inhibitors, due to which the disease eventually progresses. Compounding this issue is the fact that the acquisition of resistance is varied amongst different tumor types and thereby has not yet been characterized. In addition to the samples available for examination, there are limitations to tools available for analyses, respectively. Moreover, a uniform guideline needs to be adopted for defining acquired resistance to immunotherapy [93]. Further insight into the underlying mechanisms of acquired resistance is necessary to improve the therapeutic efficacy of current checkpoint inhibitors as well as for developing next generation of improvised inhibitors. One potential strategy to overcome this resistance or improving their therapeutic efficiency would be combining immune checkpoint inhibitors with either conventional therapies or concurrently administering two or more checkpoint inhibitors. T cell exhaustion is another limitation in the clinic. Clinical data indicated that PD1 and T cell Ig and ITIM domain (TIGIT) inhibitors regulated the expansion and function of tumor antigen-specific CD8^+^ T cell in melanoma patients [94], indicating that combining multiple checkpoint inhibitors could be a viable approach to prevent T cell exhaustion. Emerging studies show that this strategy could benefit certain rare cancers such as ovarian [95] and colorectal cancer [96].

Metabolic pathways are another attribute that can limit the efficacy of several immune-based approaches. The majority of these approaches including checkpoint inhibition, adoptive T cell therapies and oncolytic virus mediate anti-tumor responses through effector T cell responses. Many of the tumor microenvironment conditions such as low pH, reduced nutrient availability, presence of suppressive metabolites and hypoxia can impair the functioning of T cells. A recent review article beautifully summarized how the metabolic barriers impede various immunotherapeutic strategies [97].

Sterner and colleagues [98] presented a comprehensive review article on the challenges faced by CAR-T cell therapy and approaches to overcome them. In brief, one concern about the therapy is that antigens often escape. This possibly could be overcome by building dual or tandem CAR, thereby targeting multiple antigens. This strategy has shown promising results in certain cancers such as multiple myeloma [99] and B cell malignancies [100]. Another issue is restricted CAR-T cell trafficking and limited tumor infiltration. These issues could be overcome by regional delivery of the CAR-T cells. This has been successful in certain preclinical models such as mesothelin cancer [101]. CAR-T cell trafficking was demonstrated to be enhanced by through the overexpression of CXCR1/CXCR2 [102, 103]. Approaches being evaluated with the aim of improving its effectiveness in an immune-suppressed environment including combing it with PD1 inhibition [104, 105], and modulating them to secrete immunostimulatory signals like IL-12 [106] and Il-15 [107] and reducing the effects of immunosuppressive cytokines like IL-4 [108]. The other major concern surrounding this approach is the associated toxicity. CAR with modified binding affinities of scFv component [109], reduced cytokine secreting potential: CD19; B cell lymphoma [110] and genetically modified CAR: CRISPR/CAS9 mediated granulocyte macrophage colony-stimulating factor (GM-CSF) knockout CAR are being evaluated and have shown promising results in preclinical and early phase clinical trials. Further studies and clinical trials are needed to optimize and bring these approaches to clinical use for both, common and rare cancers.

Another deterrent to immune-based therapies are the high-costs associated with them. Unfortunately, not all patients who could benefit from them are able to afford them and not all costs are covered by health insurances. Similarly, receiving such advanced therapies requires consistent access to an experienced healthcare team and establishments that may be unavailable or limited in many countries with low and middle and income. Thus, based on the facts that only select individuals will benefit from these therapies and the expense and logistics associated with them, many patients either voluntarily or due to the economic burden restrict to only conventional therapies. It will be important to develop alternate approaches like biosimilars and vaccines that would be more cost-effective and globally be more accessible.

Together, these studies and reports indicate that while there are limitations to the current immune-based treatment regimes, the shortcomings have been identified and research efforts are being directed at overcoming them and improving patient outcomes.

## Conclusions and future directions

As more is discovered regarding the multiple mechanisms of action and resistance behind the various immune-based therapeutic strategies, immune therapies may become more personalized. Mechanistic details being described for these strategies are likely to help develop both improved therapeutic agents and strategies that would overcome the shortcomings of the current approaches. Combination therapies with immune therapy will likely continue to grow in the future.

For rare cancers, one form of combination therapy will continue to be administration of two variations of the same treatment, for instance two immune-checkpoint inhibitors. Some completed and some ongoing clinical trials have reported increased efficacy in treating tumors with this approach. For example as discussed in section 2.1, the combination therapy of nivolumab and ipilimumab showed significant efficacy in patients with recurrent malignant pleural mesothelioma in phase III trial, supporting its administration as the first-line of therapy [38]. Similarly, the combination therapy of nivolumab and ipilimumab has been reported to have higher efficacy than single agent nivolumab in treating sarcoma patients [40] and some biliary tract cancer patients [30]. In hepatocellular carcinoma, the combination of atezolizumab and bevacizumab generated better patient outcomes than sorafenib alone [26]. Additionally, such combinations may potentially be helpful in re-sensitizing refractory tumors. Conversely, preliminary data from some trials suggest that certain combinations could be toxic to the patients. The results from IMMUNOBIL PRODIGE 57 trial indicated that concurrent administration of paclitaxel with anti-PDL1 and anti-CTLA4 resulted in a high rate of anaphylaxis compared to the combination of taxane without anti-CTLA4 in patients with biliary tract tumors [28]. Such information is also of high value as it helps design combinations that would maximize the patient benefits and re-evaluate combinations that would be toxic.

Another combination approach is to add one form of immunotherapy to either conventional therapies or to combine multiple forms of immunotherapies together. The results from a phase II trial administering durvalumab during and after first-line chemotherapy with cisplatin and pemetrexed in patients with advanced malignant pleural mesothelioma showed promising results, supporting further trials [35]. An example of combining multiple forms of immune based approaches is the CD19-TriCAR-T therapy. This approach concurrently targets CD19 Positive Non-Hodgkin Lymphoma cells, inhibits PD-L1 signaling, and stimulates the activation and expansion of T/NK cells. CD19-TriCAR-T therapy is being evaluated in phase I (NCT03720496) and II (NCT03497533) trials [111, 112]. The success of such therapeutic strategies would potentially hold tremendous benefits. In addition to them being more effective than single agents, it theoretically would be less time consuming and more cost effective, which are also important in the management of many rare cancers.

## Data Availability

Not applicable.

## Abbreviations

CAR-T cells: Chimeric Antigen Receptor (CAR) T cell
IC: Immune checkpoint
(Treg) cells: T regulatory
PD1: Programmed Cell Death protein 1
PDL1: Programmed cell death ligand 1
CAF: Carcinoma-associated fibroblasts
HER2: Human epidermal growth factor receptor 2
MDSCs: Myeloid-derived suppressor cells
TIGIT: Ig and ITIM domain
TAMs: Tumor-associated macrophages
GEM: Genetically engineered macrophages
IFN-γ: Mouse interferon-gamma
CLDN6: Protein claudin 6
DC: Dendritic cell
PFS: Progression-free survival
LNPs: Lipid nanoparticles
ICB: Immune checkpoint blockade ()
GM-CSF: Granulocyte macrophage colony-stimulating factor
CRISPR: Clustered Regularly Interspaced Short Palindromic Repeats
Cas9: CRISPR-associated (Cas) endonuclease
